# Consumption of Commercial Non-Alcoholic Beverages Is Associated with Refractive Changes, Axial Length Changes and Myopia in Children and Adolescents

**DOI:** 10.3390/nu18142326

**Published:** 2026-07-16

**Authors:** Keyang Zheng, Dongling Yang, Xiaodong Sun, Shuangxiao Qu, Liting Chu, Shenglei Huang, Yuting Huang, Yanting Yang, Wenjuan Qi, Fengyun Zhang, Chunyan Luo

**Affiliations:** Division of Child and Adolescent Health, Shanghai Municipal Center for Disease Control and Prevention, Shanghai 200336, China; zhengkeyang@scdc.sh.cn (K.Z.); yangdongling@scdc.sh.cn (D.Y.);

**Keywords:** commercial non-alcoholic beverages, sugar-sweetened beverages, myopia, refractive error, axial length, child

## Abstract

**Background**: Myopia has become a major public health issue for the ocular health of children and adolescents worldwide and has been found to be associated with dietary factors, including the consumption of beverages. However, current research lacks detailed classification and longitudinal analysis of the relationship between different types of commercial non-alcoholic beverages and children’s visual function and related measurements. **Methods**: From 2021 to 2024, we conducted a four-year cohort study in Shanghai, China, aiming to explore the association between consumption of various commercial non-alcoholic beverages, including sugar-sweetened beverages (SSBs), non-sugar-sweetened beverages (NSSBs), and fruit and vegetable beverages, and the vision of children and adolescents. Participants were surveyed once a year, including the collection of beverage consumption frequency using a semi-quantitative food frequency questionnaire (FFQ), as well as ophthalmological examinations covering binocular uncorrected visual acuity, refraction, and axial length (AL). A total of 5199 participants with complete data were included in the study. Generalized Estimating Equations (GEE) were used to analyze the longitudinal associations between the frequency of consumption of various beverages and myopia as well as vision-related measurements. **Results**: Among the 5199 participants included in the study, 37.9% were defined as myopic at baseline, with this proportion rising to 65.6% by the end of the study in 2024. Participants with myopia typically had lower consumption frequencies of 100% and non-100% fruit and vegetable juices and higher consumption frequencies of carbonated beverages and milk tea beverages. Increased consumption of 100% and non-100% fruit and vegetable juices was associated with reduced odds of myopia, increased spherical equivalent (SE), and decreased AL in children. In contrast, increased consumption of carbonated beverages, milk tea beverages, and NSSBs was associated with higher odds of myopia, reduced SE, and increased AL in children. **Conclusions**: Different types of commercial non-alcoholic beverages have varying effects on the vision of children and adolescents. Consumption of carbonated beverages, milk tea beverages, and NSSBs is associated with myopia and AL growth in children, whereas drinking fruit and vegetable juice has been found to be associated with slower myopia progression.

## 1. Introduction

Visual impairment in children and adolescents is an increasingly serious public health problem, especially myopia [1]. Although efforts have been made globally to reduce the burden of myopia in children and adolescents, the overall prevalence has continued to rise over the past decade [2]. The trend of myopia occurring at younger ages means that children will experience a longer period of progression, leading to a higher risk of visual impairment and complications in adulthood [3,4]. Myopia in children is usually accompanied by axial eye growth and refractive errors, which are also the fundamental characteristics of early ocular lesions [5,6]. Moreover, myopia also brings a heavy economic burden to society [7].

Common commercial non-alcoholic beverages in daily life currently include sugar-sweetened beverages (SSBs), non-sugar-sweetened beverages (NSSBs), and fruit and vegetable beverages. Among them, SSBs are the main source of sugar intake for children and adolescents [8]. Although the consumption frequency of SSBs is declining in North America and Europe, the situation is not the same in developing regions such as Asia [9,10]. The World Health Organization (WHO) defines all beverages containing free sugars, including carbonated or non-carbonated drinks, flavored water made from liquid and powdered concentrates, energy drinks and sports drinks, tea beverages, coffee, and flavored milk drinks, as SSBs [11]. As an emerging type of beverage, NSSBs are experiencing rapid growth in consumption trends among children and adolescents [12,13]. Similarly, fruit and vegetable beverages are increasingly chosen as alternatives to SSB due to their nutritional value and flavor [14]. Long-term high intake of SSBs has been found to be associated with various health effects in children, including weight gain, dental caries, cardiometabolic risks, and type 2 diabetes [15,16,17,18]. A cross-sectional study of 6974 students conducted in Northeast China found that the consumption of SSBs may be associated with an increased risk of myopia in children [19]. Similarly, a cross-sectional study conducted in 14 provinces of China involving 12,397 children aged 11 to 14 found that the intake of sugary foods was associated with an increased prevalence of myopia [20]. Long-term high blood sugar has been found to damage the structure of retinal blood vessels, causing leakage, ischemia, and abnormal neovascularization [21,22]. However, the evidence regarding the impact of various beverage consumption on children’s myopia is not yet conclusive, especially in a long-term longitudinal study.

Various types of commercial non-alcoholic beverages may have different effects on eye health due to differences in their sugar content and nutritional components [23]. Therefore, we conducted a cohort study of primary and secondary school students in Shanghai, China, to investigate the effects of drinking different types of beverages on myopia in children and adolescents. In addition, we further explored the association between long-term consumption of various beverages and changes in children’s refraction and axial length (AL).

## 2. Materials and Methods

### 2.1. Study Population

We conducted a cohort study based on the Shanghai Municipal Dynamic Cohort of Student Common Diseases (SMDCSCD) to explore the association between various beverage consumptions and myopia in children and adolescents. The details of this cohort have been described in previous studies [24]. In brief, it is a prospective cohort initiated in 2021, covering 16 districts of Shanghai, China, and aims to investigate the relationship between student behaviors, environmental factors, and common diseases. A total of 6522 participants were recruited from 32 primary and secondary schools across the city, followed by annual physical examinations and questionnaire surveys. During the entire study period, 620 participants were lost to follow-up, with 576 participants (including 198 participants simultaneously missing complete vision examination data) and 127 participants excluded from the analysis due to lack of complete questionnaire data and vision examination data, respectively, leaving a total of 5199 participants included in the analysis (Figure S1). All participants and their parents/guardians have obtained informed consent before participation, and the study protocol has been approved by the Ethics Committee of the Shanghai Municipal Center for Disease Control and Prevention (“A Retrospective Study on Common Diseases and Health Influencing Factors of Students in Shanghai”, No. 2022-13).

### 2.2. Survey on Consumption of Beverages

The food frequency questionnaire (FFQ), as an effective and reliable method for dietary assessment, has been widely used in epidemiological studies on the relationship between daily diet and diseases in children and adolescents [25,26]. We used a semi-quantitative FFQ, whose reliability and validity have been verified, to investigate participants’ consumption of various beverages [27,28]. We investigated several types of commercial non-alcoholic beverages commonly encountered in daily life, including SSBs, NSSBs, and fruit and vegetable beverages. Specifically, beverages were divided into six items: ‘100% fruit and vegetable juice (including bottled and freshly squeezed),’ ‘non-100% fruit and vegetable juice (such as fresh fruit juice/orange juice/apple juice/tomato juice, etc.),’ ‘carbonated beverages (Coke/Sprite/Fanta/Mirinda/Soda, etc.),’ ‘milk tea beverages (including instant/bottled/freshly made in stores),’ ‘other sugar beverages (such as tea-flavored beverages/dairy-containing or lactic acid beverages/plant protein and grain beverages/coffee/functional beverages, etc.),’ and ‘non-sugar-sweetened beverages (labeled as sugar-free but added sweeteners, such as Coke Zero, Sprite Zero, etc.).’ Participants were asked to recall their consumption of various beverages over the past month and answer ‘Yes/No.’ If they answered ‘Yes,’ they then selected one option from ‘times/day,’ ‘times/week,’ or ‘times/month’ based on their actual drinking frequency. Finally, the drinking frequency was uniformly converted to ‘times/week’ and categorized into ‘Never,’ ‘≤1,’ ‘>1 to ≤3,’ and ‘>3.’

### 2.3. Visual Acuity Measurement and Assessment

The participants’ unaided visual acuity was measured according to the ‘Standard for Logarithmic Visual Acuity Chart’ (GB11533-2011) [29] formulated by the Standardization Administration of China. This method uses a logarithmic notation and is suitable for children and adolescents aged 3 years and older, with higher values indicating better vision. The specific measurement method has been described in previous studies [24]. In brief, participants were required to identify the direction of the symbols on the chart from a distance of 5 m in a well-lit setting (500 lx). Visual acuity in each eye was determined by the smallest row of symbols that could be correctly recognized.

The desk-mounted autorefractor (KR-8900, Topcon, Tokyo, Japan) was used to test the diopter (D) of subjects without cycloplegia, and it was calibrated using a simulated eye before testing. Each eye is measured three times, and the average value is taken. If the difference between any two measurements exceeds 0.5 D, the measurement was repeated. The accuracy of using noncycloplegic refraction (NCR) for school myopia screening has been validated [30,31]. With informed consent, some participants underwent axial length (AL) measurements throughout the study period using an ocular biometer (Carl Zeiss, IOL Master 700, Jena, Germany). Subjects are tested once a year, and the same operating procedures are followed for each examination.

Uncorrected visual acuity below 4.9 (Snellen 5/6), combined with a spherical equivalent (SE) power ≤ −0.5 D, is considered myopic. The SE was calculated as spherical (S) power + 0.5 × cylindrical (C) power. In this study, if at least one eye is defined as myopic, the subject is classified as having myopia overall.

### 2.4. Covariates

Structured questionnaires were used to collect information on basic characteristics, family, and behavioral factors of the study subjects during an annual survey. Specific information includes children’s age (years), gender (male, female), body mass index (BMI), ethnicity (Han, other), parents’ education level (primary school or below, junior high school, high school or secondary vocational, junior college, bachelor’s degree, master’s degree or above), parental myopia (yes, no), self-assessed family economic status (very good, good, fair, poor, very poor), and urban-rural type (urban, suburban). The self-report questionnaire was used to collect information related to children’s eye-use behaviors, including participation in extracurricular sports activities (yes, no), participation in extracurricular academic activities (yes, no), participation in extracurricular art activities (yes, no), the time spent on homework or reading books and magazines on school days and weekends (≤1 h/day, >1 h/day), and the time spent using electronic devices (tablets, mobile phones, etc., including for study/entertainment) on school days and weekends (≤1 h/day, >1 h/day). Outdoor activities include the time spent on school days and weekends (<2 h/day, ≥2 h/day). Information related to children’s behavior includes participation in extracurricular sports activities (yes, no), participation in learning-related extracurricular activities (yes, no), participation in art-related extracurricular activities (yes, no), time spent on homework or reading on school days and weekends (≤1 h/day, >1 h/day), and time spent using electronic devices on school days and weekends (≤1 h/day, >1 h/day). Outdoor activity time on school days and weekends (<2 h/day, ≥2 h/day).

### 2.5. Statistical Analysis

Categorical variables are reported as frequencies and percentages, and intergroup comparisons were performed using the chi-square test. Continuous variables were reported as means and standard deviations (SD), and intergroup comparisons were performed using *t*-tests.

Generalized estimating equations (GEE) were used to analyze repeated measurement data to study the longitudinal association between the frequency of various beverage consumptions and myopia, SE, and AL. The GEE model was specified with an independent correlation structure to optimize computational efficiency while avoiding unfounded assumptions about temporal or sequential dependencies and maintaining validity [32]. Time was treated as a within-subject categorical variable in the GEE model, with each year’s vision measurement considered as a repeated outcome. Participant ID was specified as the subject clustering variable in the GEE. Beverage intake and covariates for each year were used as predictor variables to assess their main effects on vision measurements. For the outcome of myopia, a binary logistic regression model using GEE was used to estimate the odds ratio (OR) of increased beverage consumption and its 95% confidence interval (CI). For SE and AL, the regression coefficient (β) and its 95% CI for increased beverage consumption were estimated using a linear regression model with GEE. Each model used the group that ‘Never’ consumed the beverage as a reference, and the results are shown as the estimated effect of increased consumption of various beverages compared to the reference group. For multiple testing, the Benjamini-Yekutieli correction was applied to control the false discovery rate (FDR).

The selection of covariates is based on the following considerations: (1) they must be independent risk factors for myopia or factors affecting visual function; (2) they must be related to the consumption of sugar-sweetened beverages and be unevenly distributed within the study population; and (3) they must not be a ‘consequence’ of consuming sugar-sweetened beverages nor an intermediate factor in the causal chain between sugar-sweetened beverage consumption and vision. All models were adjusted for the minimal sufficient and complete set of covariates identified from the directed acyclic graph (DAG) (Figure S2) we constructed based on existing literature and expert knowledge: children’s age, gender, ethnicity, body mass index (BMI), parents’ education level, household economic status, participation in extracurricular sports courses, daily outdoor activity duration on school days, outdoor activity duration on weekends, and urban-rural type.

To verify the robustness of the results, we conducted two sensitivity analyses. Firstly, based on the fully adjusted primary model, we considered the effect of parental myopia, as it is related to genetics and the family’s dietary patterns. Secondly, in the main model, we also adjusted for the potential impact of eye-use habits among children and adolescents. Their eye-use habits were assessed by combining reading and screen time on both school days and weekends.

All analyses were conducted using R (version 4.4.3; R Development Core Team, Vienna, Austria). A *p* < 0.05 for a two-tailed test was considered to indicate statistical significance.

## 3. Results

### 3.1. Participant Characteristics

Table 1 shows the baseline characteristics of the study participants. The study included 5199 participants aged 6–13 years, with an average age of 8.83 ± 2.51 years at the baseline survey, of which 2601 (49.9%) were male and 2598 (50.1%) were female, and a total of 1973 (37.9%) participants were identified as myopic. Participants defined as myopic were older (10.60 vs. 7.74 y), had higher BMIs (19.19 vs. 16.88 kg/m^2^), and a higher proportion had parents with low educational levels (*p* < 0.001). Participants with myopia had a higher proportion of fathers with myopia (51.3% vs. 48.0%), but the proportion of mothers with myopia showed no statistical difference compared with participants with normal vision. Participants with myopia have lower participation rates in extracurricular sports and art courses, higher proportions of doing homework for more than 1 h and spending less than 2 h on outdoor activities on weekends, and a lower proportion of using electronic devices for more than 1 h (all *p* < 0.001).

### 3.2. Consumption of Various Beverages by Study Participants

Table 2 shows the consumption of various beverages and the occurrence of myopia among study participants during annual follow-ups from 2021 to 2024. The proportion of myopia among participants is increasing year by year, and by 2024, a total of 3410 individuals (65.6%) were defined as myopic. For 100% fruit and vegetable juice, in the three annual surveys, the proportion of study subjects with myopia who ‘never drink’ was higher than that of those without myopia. For non-100% fruit and vegetable juice, only in the 2021 survey was the proportion of study subjects with myopia who ‘never drank’ higher than those without myopia (*p* < 0.001). For carbonated beverages, in three annual surveys, it was found that compared with those without myopia, the proportion of study subjects with myopia who ‘never drink’ was lower, and the proportion whose drinking frequency was ‘>3 times/week’ was higher. In the annual surveys conducted over four consecutive years, the proportion of study subjects with myopia who ‘never drink’ milk tea beverages was consistently lower than that of those without myopia. Observed only in the annual surveys of 2021 (*p* = 0.012) and 2024 (*p* = 0.046), compared with those without myopia, study subjects with myopia had a lower proportion of ‘never drink’ other sugary beverages and a higher proportion of those consuming them ‘>3 times/week’. For NSSBs, their consumption frequency was the lowest among the various beverages consumed by the study subjects, and in the three surveys, those with myopia were observed to consume them more frequently than those without myopia.

### 3.3. Associations Between the Consumption of Various Beverages and Myopia

The results of the generalized estimating equations showed that, using ‘never drink’ as the reference group, participants’ consumption of various beverages is associated with changes in the odds of myopia in both eyes (Table 3). For 100% fruit and vegetable juice, drinking ‘≤1 time’ [OR (95% CI) = 0.763 (0.697, 0.834)], ‘>1 to ≤3 times’ [OR (95% CI) = 0.766 (0.697, 0.843)], and ‘>3 times’ [OR (95% CI) = 0.824 (0.735, 0.922)] per week are all associated with reduced odds of myopia, and this association is statistically significant in both eyes. For non-100% fruit and vegetable juice, drinking ‘≤1 time’ [OR (95% CI) = 0.763 (0.695, 0.838)] and ‘>1 to ≤3 times’ [OR (95% CI) = 0.874 (0.777, 0.983)] per week is associated with reduced odds of myopia. For carbonated beverages, drinking ‘>3 times’ [OR (95% CI) = 1.370 (1.134, 1.654)] per week is associated with increased odds of myopia. The increased odds of myopia are associated with drinking milk tea beverages ‘≤1 time’ [OR (95% CI) = 1.488 (1.365, 1.623)] and ‘>1 to ≤3 times’ [OR (95% CI) = 1.980 (1.686, 2.324)] per week, and the association with ‘>3 times’ per week is only observed in the left eye [OR (95% CI) = 1.266 (1.022, 1.569)]. For other sugary beverages, the association between drinking ‘≤1 time’ per week and reduced odds of myopia is only observed in the left eye [OR (95% CI) = 0.916 (0.841, 0.999)], and drinking ‘>1 to ≤3 times’ per week is associated with reduced odds of overall myopia [OR (95% CI) = 0.888 (0.801, 0.986)]. Drinking NSSBs ‘>1 to ≤3 times’ per week is associated with increased odds of myopia [OR (95% CI) = 1.324 (1.085, 1.615)], which has been observed in both eyes.

### 3.4. Associations Between the Consumption of Various Beverages and Spherical Equivalent

When the SE was treated as a continuous variable, the results of the generalized estimating equations showed that participants’ consumption of various beverages was associated with changes in the SE (Table 4). For 100% fruit and vegetable juice, compared with ‘never drink,’ consuming ‘≤1 time’ [left eye β (95% CI) = 0.262 (0.178, 0.345); right eye β (95% CI) = 0.280 (0.196, 0.364)], ‘>1 to ≤3 times’ [left eye β (95% CI) = 0.274 (0.184, 0.363); right eye β (95% CI) = 0.301 (0.211, 0.391)], and ‘>3 times’ [left eye β (95% CI) = 0.231 (0.122, 0.340); right eye β (95% CI) = 0.243 (0.133, 0.352)] per week is associated with an increase in SE in both eyes. Similarly, for non-100% fruit and vegetable juice, drinking ‘≤1 time’ [left eye β (95% CI) = 0.213 (0.129, 0.298); right eye β (95% CI) = 0.245 (0.158, 0.332)] and ‘>1 to ≤3 times’ [left eye β (95% CI) = 0.172 (0.064, 0.281); right eye β (95% CI) = 0.217 (0.108, 0.326)] per week is associated with an increase in SE in both eyes. For carbonated beverages, drinking ‘>3 times’ is only associated with a decrease in the SE of the left eye [β (95% CI) = −0.221 (−0.418, −0.024)]. Compared with ‘never drink,’ drinking milk tea beverages ‘≤1 time’ [left eye β (95% CI) = −0.391 (−0.480, −0.303); right eye β (95% CI) = −0.447 (−0.535, −0.359)] and ‘>1 to ≤3 times’ [left eye β (95% CI) = −0.675 (−0.840, −0.510); right eye β (95% CI) = −0.703 (−0.874, −0.532)] per week is associated with a decrease in SE in both eyes, while the association between drinking ‘>3 times’ per week and SE is observed only in the right eye [β (95% CI) = −0.230 (−0.460, 0.000)]. For other sugary beverages, drinking ‘≤1 time’ per week is associated with an increase in SE in the right eye [β (95% CI) = 0.109 (0.025, 0.193)]. Drinking NSSBs ‘>1 to ≤3 times’ per week is associated with a decrease in the SE of both eyes [left eye β (95% CI) = −0.211 (−0.399, −0.022); right eye β (95% CI) = −0.200 (−0.386, −0.013)].

### 3.5. Associations Between the Consumption of Various Beverages and Axial Length

After adjusting for potential confounding factors, the results of the generalized estimating equations showed that participants’ consumption of various beverages was associated with changes in AL (Table 5). For 100% fruit and vegetable juice, compared with ‘never drink,’ consuming ‘≤1 time’ [left eye β (95% CI) = −0.176 (−0.242, −0.110); right eye β (95% CI) = −0.203 (−0.269, −0.138)], ‘>1 to ≤3 times’ [left eye β (95% CI) = −0.183 (−0.253, −0.113); right eye β (95% CI) = −0.195 (−0.264, −0.126)], and ‘>3 times’ [left eye β (95% CI) = −0.096 (−0.181, −0.012); right eye β (95% CI) = −0.105 (−0.189, −0.021)] per week is negatively associated with the AL of both eyes. Consistently, for non-100% fruit and vegetable juice, drinking ‘≤1 time’ [left eye β (95% CI) = −0.184 (−0.251, −0.117); right eye β (95% CI) = −0.191 (−0.259, −0.124)] and ‘>1 to ≤3 times’ [left eye β (95% CI) = −0.129 (−0.213, −0.045); right eye β (95% CI) = −0.119 (−0.204, −0.034)] per week is negatively associated with the AL of both eyes. For carbonated beverages, drinking ‘>1 to ≤3 times’ [left eye β (95% CI) = 0.226 (0.130, 0.322); right eye β (95% CI) = 0.235 (0.135, 0.335)] and ‘>3 times’ [left eye β (95% CI) = 0.380 (0.248, 0.513); right eye β (95% CI) = 0.375 (0.244, 0.505)] per week is positively associated with the AL of both eyes. Drinking milk tea beverages ‘≤1 time’ [left eye β (95% CI) = 0.155 (0.091, 0.219); right eye β (95% CI) = 0.170 (0.106, 0.234)] and ‘>1 to ≤3 times’ [left eye β (95% CI) = 0.310 (0.194, 0.427); right eye β (95% CI) = 0.303 (0.183, 0.423)] per week is positively associated with the AL of both eyes. No statistical association was observed between the consumption of other sugary beverages and AL. For NSSBs, a positive association was observed between drinking ‘>1 to ≤3 times’ per week and AL of the right eye [β (95% CI) = 0.143 (0.001, 0.286)].

### 3.6. Sensitivity Analysis

Through a series of sensitivity analyses, the robustness of our main results was verified (Tables S1–S6). Regardless of whether we adjusted for parental myopia or the children’s visual habits, the overall direction of all results remained consistent with the fully adjusted model. Only a few results in the primary models, where the *p*-values were at the threshold, lost or gained statistical significance after adjusting for additional covariates. Specifically, after adjusting for parental myopia, the association between participants’ beverage consumption and vision measurements appeared to be strengthened, whereas after adjusting for children’s visual habits, these effects seemed to be attenuated. Furthermore, we found that parental myopia was associated with increased odds of myopia and SE decline in children (Tables S1 and S2). Paternal myopia was positively associated with AL, while maternal myopia showed no such association (Table S3). We also found that children’s daily reading and screen time were associated with increased odds of myopia, decreased SE, and AL growth (Tables S4–S6).

## 4. Discussion

This four-year cohort study conducted in Shanghai, China, examined the association between the consumption of various commercial non-alcoholic beverages and vision in children and adolescents. We observed that participants defined as having myopia generally consumed ‘100% and non-100% fruit and vegetable juices’ less frequently, while they consumed ‘carbonated beverages’ and ‘milk tea beverages’ more frequently. In addition, increased frequency of drinking ‘100% and non-100% fruit and vegetable juices’ was associated with reduced odds of myopia, increased SE, and decreased AL, whereas the same effect of drinking ‘other sugar beverages’ was only observed in a single eye. Increased frequency of consuming ‘carbonated beverages,’ ‘milk tea beverages,’ and ‘NSSBs’ was associated with increased odds of myopia, decreased SE, and increased AL. In addition, parental myopia and prolonged near-work habits have also been found to be associated with adverse changes in vision among children and adolescents.

As far as we know, this is the first longitudinal study to explore the relationship between children and adolescents consuming beverages and vision and its related measurements. Consistent with our findings, a cross-sectional study conducted among primary and secondary school students in Northeast China on myopia and dietary factors found that fruit intake was associated with reduced odds of myopia, while the consumption of SSBs was associated with increased odds of myopia [19]. Although this previous study investigated a wider variety of foods, it only focused on SSBs as a whole category without further subdivision. Our study expands the existing findings, indicating that among various types of beverages, the potential contributions to myopia may be attributed to carbonated drinks, milk tea beverages, and sweetened drinks. Consumption of fruit and vegetable beverages may be associated with a slower progression of myopia in children and adolescents.

The differential effects of various types of beverages on the vision of children and adolescents observed in this study can be partially explained by differences in their nutritional components and physiological mechanisms. The World Health Organization recommends reducing the intake of free sugars to less than 10% or even below 5% of total energy intake, which basically corresponds to the Chinese Dietary Guidelines (2022) recommendation that daily added sugar intake should not exceed 50 g, and it is best to control it below 25 g [11,33]. A commonly sold 500 mL bottle of carbonated drinks or milk tea usually contains about 50 g of sugar, so drinking a bottle exceeds the recommended daily sugar intake limit. Physiologically, excessive sugar intake interferes with calcium metabolism in the body, combines with calcium, and is excreted from the body, leading to a decrease in blood calcium levels [34]. Calcium is not only a regulator of signals for the ciliary muscle and retina sclera but also a key element in maintaining scleral elasticity and strength [35,36]. When the sclera of the passage is insufficiently tough, the eyeball is easily elongated due to the long-term effect of intraocular pressure, which in turn leads to axial myopia [37]. High sugar intake can also cause an imbalance in the aqueous humor and lens osmotic pressure by elevating blood sugar, leading to lens protrusion, and long-term repeated occurrences can exacerbate ocular accommodation dysfunction [38,39]. In addition, a large amount of vitamin B1 is consumed during sugar metabolism, and vitamin B1 is an important nutritional source for the optic nerve [40]. A deficiency in vitamin B1 can lead to impaired optic nerve function, thereby exacerbating visual fatigue and vision decline [41,42].

In contrast, 100% and non-100% fruit and vegetable juices have sugars that are mainly naturally occurring fructose and have relatively low sugar content [43,44]. Moreover, fruit and vegetable juices are rich in eye-protective nutrients such as vitamin A, lutein, and zinc, which play a positive role in maintaining retinal health and alleviating visual fatigue [45,46,47]. Among them, lutein, as the core pigment of the macular region of the retina, can filter blue light and exert antioxidant effects, protecting photoreceptor cells [48]. Vitamin A is involved in the synthesis of rhodopsin and is crucial for maintaining the health of the cornea and retina [49]. The presence of these nutrients may offset or mitigate some of the adverse effects of sugar on vision, resulting in research findings associated with reduced odds of myopia in children. It is worth noting that in this study, the protective effect of ‘other sugary beverages’ on vision was observed only in one eye, which may be related to individual differences in binocular refractive development, slight fluctuations in drinking frequency, or measurement errors and needs to be further verified in future research.

For NSSBs, non-sugar sweeteners (NSS) are used as substitutes for sugar [50]. Although NSS can achieve dietary balance by controlling blood sugar and reducing energy intake, its health effects remain controversial [51]. Long-term use of NSS has been found to be associated with various adverse outcomes, including effects on the nervous system, metabolic risks, alterations in the gut microbiota, and inflammation [52]. We hypothesize that the metabolic disturbances and inflammatory responses caused by NSS may indirectly affect visual function in children. An animal study indicated that high exposure to sucralose (SUC) was associated with the remodeling of diurnal tear rhythms, reductions in metabolic and immune pathway characteristics, and decreased tear secretion in mice [53]. The disruption of lacrimal gland function may be related to corneal sensory nerve damage through alterations in the circadian rhythm of mice [54]. In addition, aspartame, the mainstream artificial sweetener in NSSBs, can increase free calcium within retinal neurons through multiple pathways, and dysregulation of retinal calcium signaling is a core upstream pathway regulating axial eye growth and refractive shifts [55]. However, there is a lack of epidemiological evidence linking NSSBs with the occurrence of myopia in children, and their active effect components should be further investigated in the future.

The biological mechanisms underlying the potential association between beverages and visual function in children remain unclear. Based on the existing evidence, we provide some hypotheses. Specifically, long-term excessive consumption of SSBs can lead to abnormally elevated levels of insulin-like growth factor-1 (IGF-1) in the body [56]. IGF-1, as a key cytokine regulating the growth and development of the eyeball, its overexpression can stimulate the proliferation of scleral fibroblasts and collagen remodeling, causing the scleral wall to thin and lose elasticity, thereby promoting abnormal axial elongation and ultimately inducing or exacerbating axial myopia [57,58]. The association between IGF1 single nucleotide polymorphisms and myopia in Chinese children has been reported [59]. On the other hand, high sugar intake can also trigger ocular inflammatory responses by activating inflammatory pathways in the body [60,61]. Advanced glycation end products (AGEs) produced by excessive sugar metabolism accumulate in retinal tissues and induce retinal pigment epithelial cells to release inflammatory factors (such as TNF-α and IL-6) [62,63]. These inflammatory factors can damage retinal photoreceptor cells and choroidal blood vessels, disrupt the function of the retinal barrier, lead to retinal dysfunction, and, consequently, affect visual acuity and visual quality [64,65]. Clarifying these mechanisms will help to more comprehensively understand the relationship between SSB consumption and myopia in children.

Although this study has significant theoretical and practical value, there are still some limitations that need to be acknowledged. First, this study is a cohort study, which can reveal the association between various beverage consumptions and the vision of children and adolescents, but it cannot fully establish a causal relationship. It is worth noting that this study has controlled for various potential confounding factors to minimize interference as much as possible, but it still cannot completely rule out the influence of potential confounding factors that were not included, such as differences in individual genetic susceptibility and baseline eye health. Secondly, the subjects of this study were only from the Shanghai area in China. As an economically developed city, the dietary structure and lifestyle habits of children and adolescents in Shanghai may differ from those in inland or rural areas, so the generalizability of the study results may be somewhat limited. Thirdly, our refractive measurements were conducted in a non-cycloplegic state, which may affect the assessment of myopia. Nevertheless, previous studies have validated the accuracy of NCR for large-scale myopia screening [30,31]. Finally, the classification of various beverages in this study was mainly based on beverage type and did not further refine the differences in their composition, such as the sugar concentration in different brands of carbonated beverages, the creamer in milk tea beverages, or the content of additives. These detailed differences may have different effects on vision. In addition, the assessment of the frequency of various beverage intakes in the study relies on participants’ self-reports, which may be subject to recall bias, and it is not possible to determine the actual intake of nutrients such as total sugar and total energy by the participants.

## 5. Conclusions

In conclusion, through a four-year longitudinal cohort study, this research has, for the first time, clearly identified the differentiated associations between various types of commercial non-alcoholic beverages and vision as well as related measurement indicators in children and adolescents, expanding the existing evidence on the relationship between diet and myopia. The study results indicate that carbonated beverages, consumed on average more than three times per week, as well as milk tea and NSSBs, consumed on average one to three times per week, may be associated with the odds of myopia, refractive errors, and AL growth in children and adolescents. Consumption of fruit and vegetable juices on average less than three times per week was found to potentially be associated with a slowing of myopia. Further in-depth studies are needed in the future to support the advancement of myopia prevention and control efforts.

## Figures and Tables

**Table 1:** Baseline characteristics of study participants included in the analysis in the SMDCSCD cohort.

Characteristics	Total (n = 5199)	Myopia (n = 1973)	Non-Myopia (n = 3226)	*p*-Value ^a^
Age [y, mean (SD)]	8.83 (2.51)	10.60 (2.05)	7.74 (2.11)	<0.001
Gender [n (%)]				0.170
Male	2601 (49.9)	963 (48.8)	1638 (50.8)	
Female	2598 (50.1)	1010 (51.2)	1588 (49.2)	
BMI [kg/m^2^, mean (SD)]	17.76 (3.69)	19.19 (3.97)	16.88 (3.23)	<0.001
Ethnicity [n (%)]				0.128
Han	5121 (98.5)	1950 (98.8)	3171 (98.3)	
Other	78 (1.5)	23 (1.2)	55 (1.7)	
Father’s education [n (%)]				<0.001
Primary school and below	37 (0.7)	17 (0.9)	20 (0.6)	
Junior high school	373 (7.5)	185 (9.4)	188 (5.8)	
High school or secondary vocational	757 (14.6)	327 (16.6)	430 (13.3)	
Junior college	1309 (25.2)	536 (27.2)	773 (24.0)	
Bachelor’s degree	2173 (41.8)	731 (37.1)	1442 (44.7)	
Master’s degree and above	550 (10.6)	177 (9.0)	373 (11.6)	
Mother’s education [n (%)]				<0.001
Primary school and below	77 (1.5)	39 (2.0)	38 (1.2)	
Junior high school	432 (8.3)	201 (10.2)	231 (7.2)	
High school or secondary vocational	658 (12.7)	302 (15.3)	356 (11.0)	
Junior college	1361 (26.2)	540 (27.4)	821 (25.4)	
Bachelor’s degree	2318 (44.6)	778 (39.4)	1540 (47.7)	
Master’s degree and above	353 (6.8)	113 (5.7)	240 (7.4)	
Father’s myopia				0.022
Yes	2561 (49.3)	1012 (51.3)	1549 (48.0)	
No	2638 (50.7)	961 (48.7)	1677 (52.0)	
Mother’s myopia				0.529
Yes	2768 (53.2)	1039 (52.7)	1729 (53.6)	
No	2431 (46.8)	934 (47.3)	1497 (46.4)	
Household economic status [n (%)]				<0.001
Very good	234 (4.5)	122 (6.2)	112 (3.5)	
Good	1430 (27.5)	592 (30.0)	838 (26.0)	
Fair	3435 (66.1)	1223 (62.0)	2212 (68.6)	
Poor	82 (1.6)	28 (1.4)	54 (1.7)	
Very poor	18 (0.3)	8 (0.4)	10 (0.3)	
Urban-rural type [n (%)]				0.184
Urban	2205 (42.4)	860 (43.6)	1345 (41.7)	
Suburban	2994 (57.6)	1113 (56.4)	1881 (58.3)	
Extracurricular sports courses				<0.001
Yes	2621 (50.6)	832 (42.2)	1789 (55.5)	
No	2578 (49.6)	1141 (57.8)	1437 (44.5)	
Extracurricular learning courses				0.858
Yes	1842 (35.4)	702 (35.6)	1140 (35.3)	
No	3357 (64.6)	1271 (64.4)	2086 (64.7)	
Extracurricular art courses				<0.001
Yes	2661 (51.2)	846 (42.9)	1815 (56.3)	
No	2538 (48.8)	1127 (57.1)	1411 (43.7)	
Study day reading time				<0.001
≤1 h	2078 (40.0)	572 (29.0)	1506 (46.7)	
>1 h	3121 (60.0)	1401 (71.0)	1720 (53.3)	
Weekend reading time				<0.001
≤1 h	1643 (31.6)	389 (19.7)	1254 (38.9)	
>1 h	3556 (68.4)	1584 (80.3)	1972 (61.1)	
Time spent on electronics on study days				<0.001
≤1 h	4340 (83.5)	1693 (85.8)	2647 (82.1)	
>1 h	859 (16.5)	280 (14.2)	579 (17.9)	
Time spent on electronics on weekends				<0.001
≤1 h	3075 (59.1)	1058 (53.6)	2017 (62.5)	
>1 h	2124 (40.9)	915 (46.4)	1209 (37.5)	
Outdoor activity time on study days				0.398
<2 h	4728 (90.9)	1803 (91.4)	2925 (90.7)	
≥2 h	471 (9.1)	170 (8.6)	301 (9.3)	
Outdoor activity time on weekends				<0.001
<2 h	2106 (40.5)	919 (46.6)	1187 (36.8)	
≥2 h	3093 (59.5)	1054 (53.4)	2039 (63.2)	

Note: BMI, body mass index; SD, standard deviation; h, hour. ^a^: The *p*-values were calculated by chi-square test (categorical variable) or independent-samples *t*-test (a normally distributed continuous variable).

**Table 2:** Frequency of consumption of various beverages among study participants grouped by myopia from 2021 to 2024.

Beverage Type (Times/Week)	2021				2022				2023				2024			
	N (%)	n ^a^ (%)	n ^b^ (%)	*p* ^c^	N (%)	n ^a^ (%)	n ^b^ (%)	*p* ^c^	N (%)	n ^a^ (%)	n ^b^ (%)	*p* ^c^	N (%)	n ^a^ (%)	n ^b^ (%)	*p* ^c^
100% fruit and vegetable juice	5199 (100)	1973 (100)	3226 (100)	<0.001	5199 (100)	2554 (100)	2645 (100)	0.050	5199 (100)	2967 (100)	2232 (100)	0.038	5199 (100)	3410 (100)	1789 (100)	0.074
Never	2392 (46.0)	981 (49.7)	1411 (43.7)		3180 (61.2)	1600 (62.6)	1580 (59.7)		3252 (62.6)	1893 (63.8)	1359 (60.9)		3142 (60.4)	2101 (61.6)	1041 (58.2)	
≤1	1257 (24.2)	405 (20.5)	852 (26.4)		750 (14.4)	355 (13.9)	395 (14.9)		738 (14.2)	420 (14.2)	318 (14.2)		799 (15.4)	501 (14.7)	298 (16.7)	
>1 to ≤3	880 (16.9)	317 (16.1)	563 (17.5)		791 (15.2)	358 (14.0)	433 (16.4)		719 (13.8)	376 (12.7)	343 (15.4)		716 (13.8)	453 (13.3)	263 (14.7)	
>3	670 (12.9)	270 (13.7)	400 (12.4)		478 (9.2)	241 (9.4)	237 (9.0)		490 (9.4)	278 (9.4)	212 (9.5)		542 (10.4)	355 (10.4)	187 (10.5)	
Non-100% fruit and vegetable juice				<0.001				0.221				0.062				0.113
Never	3117 (60.0)	1217 (61.7)	1900 (58.9)		3856 (74.2)	1887 (73.9)	1969 (74.4)		3896 (74.9)	2256 (76.0)	1640 (73.5)		3600 (69.2)	2392 (70.1)	1208 (67.5)	
≤1	1293 (24.9)	425 (21.5)	868 (26.9)		752 (14.5)	357 (14.0)	395 (14.9)		705 (13.6)	371 (12.5)	334 (15.0)		813 (15.6)	513 (15.0)	300 (16.8)	
>1 to ≤3	515 (9.9)	203 (10.3)	312 (9.7)		403 (7.8)	206 (8.1)	197 (7.4)		396 (7.6)	221 (7.4)	175 (7.8)		464 (8.9)	289 (8.5)	175 (9.8)	
>3	274 (5.3)	128 (6.5)	146 (4.5)		188 (3.6)	104 (4.1)	84 (3.2)		202 (3.9)	119 (4.0)	83 (3.7)		322 (6.2)	216 (6.3)	106 (5.9)	
Carbonated beverages				<0.001				<0.001				0.193				0.005
Never	3183 (61.2)	1126 (57.1)	2057 (63.8)		3620 (69.6)	1705 (66.8)	1915 (72.4)		3597 (69.2)	2034 (68.6)	1563 (70.0)		3262 (62.7)	2142 (62.8)	1120 (62.6)	
≤1	1451 (27.9)	583 (29.5)	868 (26.9)		1069 (20.6)	556 (21.8)	513 (19.4)		1014 (19.5)	579 (19.5)	435 (19.5)		1150 (22.1)	737 (21.6)	413 (23.1)	
>1 to ≤3	377 (7.3)	170 (8.6)	207 (6.4)		388 (7.5)	212 (8.3)	176 (6.7)		426 (8.2)	264 (8.9)	162 (7.3)		512 (9.8)	325 (9.5)	187 (10.5)	
>3	188 (3.6)	94 (4.8)	94 (2.9)		122 (2.3)	81 (3.2)	41 (1.6)		162 (3.1)	90 (3.0)	72 (3.2)		275 (5.3)	206 (6.0)	69 (3.9)	
Milk tea beverages				<0.001				<0.001				0.007				<0.001
Never	3440 (66.2)	1170 (59.3)	2270 (70.4)		3701 (71.2)	1719 (67.3)	1982 (74.9)		3644 (70.1)	2029 (68.4)	1615 (72.4)		3252 (62.6)	2075 (60.9)	1177 (65.8)	
≤1	1422 (27.4)	626 (31.7)	796 (24.7)		1185 (22.8)	645 (25.3)	540 (20.4)		1204 (23.2)	717 (24.2)	487 (21.8)		1481 (28.5)	996 (29.2)	485 (27.1)	
>1 to ≤3	205 (3.9)	108 (5.5)	97 (3.0)		208 (4.0)	134 (5.2)	74 (2.8)		221 (4.3)	144 (4.9)	77 (3.4)		308 (5.9)	231 (6.8)	77 (4.3)	
>3	132 (2.5)	69 (3.5)	63 (2.0)		105 (2.0)	56 (2.2)	49 (1.9)		130 (2.5)	77 (2.6)	53 (2.4)		158 (3.0)	108 (3.2)	50 (2.8)	
Other sugary beverages ^d^				0.012				0.507				0.520				0.046
Never	2540 (48.9)	940 (47.6)	1600 (49.6)		3409 (65.6)	1661 (65.0)	1748 (66.1)		3423 (65.8)	1948 (65.7)	1475 (66.1)		3045 (58.6)	1968 (57.7)	1077 (60.2)	
≤1	1522 (29.3)	571 (28.9)	951 (29.5)		998 (19.2)	503 (19.7)	495 (18.7)		1004 (19.3)	561 (18.9)	443 (19.8)		1202 (23.1)	784 (23.0)	418 (23.4)	
>1 to ≤3	721 (13.9)	273 (13.8)	448 (13.9)		536 (10.3)	256 (10.0)	280 (10.6)		517 (9.9)	308 (10.4)	209 (9.4)		624 (12.0)	423 (12.4)	201 (11.2)	
>3	416 (8.0)	189 (9.6)	227 (7.0)		256 (4.9)	134 (5.2)	122 (4.6)		255 (4.9)	150 (5.1)	105 (4.7)		328 (6.3)	235 (6.9)	93 (5.2)	
Non-sugar-sweetened beverages ^e^				<0.001				0.014				0.354				0.004
Never	4210 (81.0)	1525 (77.3)	2685 (83.2)		4565 (87.8)	2215 (86.7)	2350 (88.8)		4564 (87.8)	2601 (87.7)	1963 (87.9)		4330 (83.3)	2843 (83.4)	1487 (83.1)	
≤1	739 (14.2)	320 (16.2)	419 (13.0)		460 (8.8)	234 (9.2)	226 (8.5)		458 (8.8)	259 (8.7)	199 (8.9)		588 (11.3)	360 (10.6)	228 (12.7)	
>1 to ≤3	147 (2.8)	72 (3.6)	75 (2.3)		112 (2.2)	70 (2.7)	42 (1.6)		111 (2.1)	72 (2.4)	39 (1.7)		151 (2.9)	109 (3.2)	42 (2.3)	
>3	103 (2.0)	56 (2.8)	47 (1.5)		62 (1.2)	35 (1.4)	27 (1.0)		66 (1.3)	35 (1.2)	31 (1.4)		130 (2.5)	98 (2.9)	32 (1.8)	

Note: The data show the consumption of various beverages within the month prior to the survey date. ^a^ is the myopia group; ^b^ is the non-myopia group. ^c^: The *p*-values were calculated using the chi-square test. ^d^: Includes tea-flavored beverages, dairy-containing or lactic acid bacteria beverages, plant protein and grain beverages, coffee, and functional beverages. ^e^: Drinks labeled as sugar-free but with added sweeteners.

**Table 3:** Generalized estimating equations analysis of the frequency of consumption of various beverages and myopia among research participants from 2021 to 2024 (n = 5199).

Beverage Type	Drinking Frequency (Times/Week)	Left Eye		Right Eye		Overall	
		OR (95% CI)	*p*	OR (95% CI)	*p*	OR (95% CI)	*p*
100% fruit and vegetable juice	Never	Ref	-	Ref	-	Ref	-
	≤1	0.791 (0.723, 0.865)	<0.001	0.759 (0.693, 0.83)	<0.001	0.763 (0.697, 0.834)	<0.001
	>1 to ≤3	0.794 (0.722, 0.873)	<0.001	0.756 (0.687, 0.832)	<0.001	0.766 (0.697, 0.843)	<0.001
	>3	0.867 (0.773, 0.973)	0.015	0.835 (0.745, 0.936)	0.002	0.824 (0.735, 0.922)	0.001
Non-100% fruit and vegetable juice	Never	Ref	-	Ref	-	Ref	-
	≤1	0.815 (0.741, 0.895)	<0.001	0.766 (0.697, 0.842)	<0.001	0.763 (0.695, 0.838)	<0.001
	>1 to ≤3	0.860 (0.765, 0.968)	0.012	0.848 (0.755, 0.953)	0.006	0.874 (0.777, 0.983)	0.024
	>3	1.012 (0.862, 1.188)	0.885	1.036 (0.883, 1.217)	0.663	1.046 (0.89, 1.229)	0.584
Carbonated beverages	Never	Ref	-	Ref	-	Ref	-
	≤1	1.052 (0.961, 1.152)	0.272	1.085 (0.992, 1.187)	0.075	1.085 (0.992, 1.187)	0.074
	>1 to ≤3	1.092 (0.962, 1.240)	0.173	1.115 (0.982, 1.266)	0.092	1.116 (0.982, 1.269)	0.092
	>3	1.228 (1.017, 1.483)	0.033	1.325 (1.098, 1.598)	0.003	1.370 (1.134, 1.654)	0.001
Milk tea beverages	Never	Ref	-	Ref	-	Ref	-
	≤1	1.468 (1.346, 1.601)	<0.001	1.461 (1.340, 1.593)	<0.001	1.488 (1.365, 1.623)	<0.001
	>1 to ≤3	1.944 (1.663, 2.271)	<0.001	1.948 (1.660, 2.286)	<0.001	1.980 (1.686, 2.324)	<0.001
	>3	1.266 (1.022, 1.569)	0.031	1.159 (0.938, 1.433)	0.171	1.222 (0.989, 1.511)	0.064
Other sugary beverages ^a^	Never	Ref	-	Ref	-	Ref	-
	≤1	0.916 (0.841, 0.999)	0.047	0.959 (0.881, 1.045)	0.338	0.920 (0.844, 1.003)	0.057
	>1 to ≤3	0.911 (0.821, 1.011)	0.081	0.920 (0.830, 1.021)	0.117	0.888 (0.801, 0.986)	0.025
	>3	1.002 (0.873, 1.150)	0.977	0.973 (0.848, 1.116)	0.698	0.972 (0.848, 1.115)	0.689
Non-sugar-sweetened beverages ^b^	Never	Ref	-	Ref	-	Ref	-
	≤1	0.994 (0.891, 1.110)	0.921	0.989 (0.887, 1.102)	0.839	0.995 (0.893, 1.109)	0.926
	>1 to ≤3	1.225 (1.007, 1.491)	0.043	1.291 (1.054, 1.580)	0.014	1.324 (1.085, 1.615)	0.006
	>3	1.091 (0.846, 1.406)	0.501	1.174 (0.910, 1.513)	0.217	1.133 (0.879, 1.461)	0.334

Note: OR, odds ratio; CI, confidence interval; Ref, reference. The data show the consumption of various beverages within the month prior to the survey date. The model adjusted for children’s age, gender, ethnicity, body mass index (BMI), parents’ education level, household economic status, participation in extracurricular sports courses, daily outdoor activity duration on school days, outdoor activity duration on weekends, and urban-rural type. ^a^: Includes tea-flavored beverages, dairy-containing or lactic acid bacteria beverages, plant protein and grain beverages, coffee, and functional beverages. ^b^: Drinks labeled as sugar-free but with added sweeteners.

**Table 4:** Generalized estimating equations analysis of the frequency of consumption of various beverages and spherical equivalent (SE) among research participants from 2021 to 2024 (n = 5199).

Beverage Type	Drinking Frequency (Times/Week)	Left Eye		Right Eye	
		β (95% CI)	*p*	β (95% CI)	*p*
100% fruit and vegetable juice	Never	Ref	-	Ref	-
	≤1	0.262 (0.178, 0.345)	<0.001	0.280 (0.196, 0.364)	<0.001
	>1 to ≤3	0.274 (0.184, 0.363)	<0.001	0.301 (0.211, 0.391)	<0.001
	>3	0.231 (0.122, 0.340)	<0.001	0.243 (0.133, 0.352)	<0.001
Non-100% fruit and vegetable juice	Never	Ref	-	Ref	-
	≤1	0.213 (0.129, 0.298)	<0.001	0.245 (0.158, 0.332)	<0.001
	>1 to ≤3	0.172 (0.064, 0.281)	0.002	0.217 (0.108, 0.326)	<0.001
	>3	0.002 (−0.152, 0.156)	0.980	−0.003 (−0.157, 0.151)	0.973
Carbonated beverages	Never	Ref	-	Ref	-
	≤1	−0.017 (−0.106, 0.072)	0.701	−0.031 (−0.120, 0.059)	0.501
	>1 to ≤3	−0.06 (−0.191, 0.070)	0.365	−0.098 (−0.231, 0.035)	0.148
	>3	−0.221 (−0.418, −0.024)	0.028	−0.183 (−0.380, 0.014)	0.068
Milk tea beverages	Never	Ref	-	Ref	-
	≤1	−0.391 (−0.480, −0.303)	<0.001	−0.447 (−0.535, −0.359)	<0.001
	>1 to ≤3	−0.675 (−0.840, −0.510)	<0.001	−0.703 (−0.874, −0.532)	<0.001
	>3	−0.214 (−0.439, 0.011)	0.063	−0.230 (−0.460, 0.000)	0.050
Other sugary beverages ^a^	Never	Ref	-	Ref	-
	≤1	0.076 (−0.006, 0.159)	0.071	0.109 (0.025, 0.193)	0.011
	>1 to ≤3	0.065 (−0.035, 0.165)	0.203	0.110 (0.009, 0.210)	0.032
	>3	−0.065 (−0.203, 0.074)	0.361	−0.047 (−0.188, 0.093)	0.508
Non-sugar-sweetened beverages ^b^	Never	Ref	-	Ref	-
	≤1	−0.006 (−0.111, 0.100)	0.916	−0.005 (−0.112, 0.102)	0.923
	>1 to ≤3	−0.211 (−0.399, −0.022)	0.029	−0.200 (−0.386, −0.013)	0.036
	>3	−0.180 (−0.451, 0.090)	0.192	−0.233 (−0.508, 0.042)	0.096

Note: β, regression coefficient; CI, confidence interval; Ref, reference. The data show the consumption of various beverages within the month prior to the survey date. The model adjusted for children’s age, gender, ethnicity, body mass index (BMI), parents’ education level, household economic status, participation in extracurricular sports courses, daily outdoor activity duration on school days, outdoor activity duration on weekends, and urban-rural type. ^a^: Includes tea-flavored beverages, dairy-containing or lactic acid bacteria beverages, plant protein and grain beverages, coffee, and functional beverages. ^b^: Drinks labeled as sugar-free but with added sweeteners.

**Table 5:** Generalized estimating equations analysis of the frequency of consumption of various beverages and axial length (AL) among research participants from 2021 to 2024 (n = 4751).

Beverage Type	Drinking Frequency (Times/Week)	Left Eye		Right Eye	
		β (95% CI)	*p*	β (95% CI)	*p*
100% fruit and vegetable juice	Never	Ref	-	Ref	-
	≤1	−0.176 (−0.242, −0.110)	<0.001	−0.203 (−0.269, −0.138)	<0.001
	>1 to ≤3	−0.183 (−0.253, −0.113)	<0.001	−0.195 (−0.264, −0.126)	<0.001
	>3	−0.096 (−0.181, −0.012)	0.026	−0.105 (−0.189, −0.021)	0.014
Non-100% fruit and vegetable juice	Never	Ref	-	Ref	-
	≤1	−0.184 (−0.251, −0.117)	<0.001	−0.191 (−0.259, −0.124)	<0.001
	>1 to ≤3	−0.129 (−0.213, −0.045)	0.003	−0.119 (−0.204, −0.034)	0.006
	>3	−0.071 (−0.187, 0.046)	0.235	−0.069 (−0.185, 0.046)	0.240
Carbonated beverages	Never	Ref	-	Ref	-
	≤1	0.055 (−0.011, 0.122)	0.103	0.047 (−0.020, 0.113)	0.168
	>1 to ≤3	0.226 (0.130, 0.322)	<0.001	0.235 (0.135, 0.335)	<0.001
	>3	0.380 (0.248, 0.513)	<0.001	0.375 (0.244, 0.505)	<0.001
Milk tea beverages	Never	Ref	-	Ref	-
	≤1	0.155 (0.091, 0.219)	<0.001	0.170 (0.106, 0.234)	<0.001
	>1 to ≤3	0.310 (0.194, 0.427)	<0.001	0.303 (0.183, 0.423)	<0.001
	>3	−0.032 (−0.205, 0.141)	0.718	−0.046 (−0.218, 0.127)	0.602
Other sugary beverages ^a^	Never	Ref	-	Ref	-
	≤1	−0.035 (−0.097, 0.027)	0.270	−0.046 (−0.109, 0.017)	0.152
	>1 to ≤3	0.008 (−0.071, 0.088)	0.837	0.007 (−0.073, 0.087)	0.863
	>3	0.056 (−0.043, 0.155)	0.269	0.028 (−0.072, 0.127)	0.587
Non-sugar-sweetened beverages ^b^	Never	Ref	-	Ref	-
	≤1	0.054 (−0.025, 0.134)	0.182	0.064 (−0.015, 0.143)	0.112
	>1 to ≤3	0.126 (−0.014, 0.265)	0.079	0.143 (0.001, 0.286)	0.048
	>3	0.162 (−0.031, 0.354)	0.099	0.187 (−0.006, 0.380)	0.057

Note: β, regression coefficient; CI, confidence interval; Ref, reference. The data show the consumption of various beverages within the month prior to the survey date. The model adjusted for children’s age, gender, ethnicity, body mass index (BMI), parents’ education level, household economic status, participation in extracurricular sports courses, daily outdoor activity duration on school days, outdoor activity duration on weekends, and urban-rural type. ^a^: Includes tea-flavored beverages, dairy-containing or lactic acid bacteria beverages, plant protein and grain beverages, coffee, and functional beverages. ^b^: Drinks labeled as sugar-free but with added sweeteners.

## Data Availability

The data used in this study are not publicly available due to restrictions related to participant privacy, institutional regulations, and data-sharing agreements. Access to the data is limited to the research team and requires approval from the relevant ethics committees and data custodians.

## Supplementary Materials

The following supporting information can be downloaded at: https://www.mdpi.com/article/10.3390/nu18142326/s1, Table S1: Generalized estimating equations analysis of the frequency of consumption of various beverages and myopia among research participants from 2021 to 2024 (n = 5199) after adjusting for parental myopia; Table S2: Generalized estimating equations analysis of the frequency of consumption of various beverages and spherical equivalent (SE) among research participants from 2021 to 2024 (n = 5199) after adjusting for parental myopia; Table S3: Generalized estimating equations analysis of the frequency of consumption of various beverages and axial length (AL) among research participants from 2021 to 2024 (n = 4751) after adjusting for parental myopia; Table S4: Generalized estimating equations analysis of the frequency of consumption of various beverages and myopia among research participants from 2021 to 2024 (n = 5199) after adjusting for eye-use habits; Table S5: Generalized estimating equations analysis of the frequency of consumption of various beverages and spherical equivalent (SE) among research participants from 2021 to 2024 (n = 5199) after adjusting for eye-use habits; Table S6: Generalized estimating equations analysis of the frequency of consumption of various beverages and axial length (AL) among research participants from 2021 to 2024 (n = 4751) after adjusting for eye-use habits; Figure S1: Flowchart for the inclusion of students from Shanghai 16 districts in this study; Figure S2: Directed acyclic graph for the association between sugar-sweetened beverages and visual impairment in children.

## Abbreviations

SSBs	sugar-sweetened beverages
NSSBs	non-sugar-sweetened beverages
FFQ	food frequency questionnaire
NCR	noncycloplegic refraction
AL	axial length
GEE	generalized estimating equations
SE	spherical equivalent
FDR	false discovery rate
WHO	World Health Organization
SD	standard deviations
OR	odds ratio
CI	confidence interval
DAG	directed acyclic graph
BMI	body mass index
NSS	non-sugar sweeteners
SUC	sucralose
