# Supplementary material for: Consumption of Commercial Non-Alcoholic Beverages Is Associated with Refractive Changes, Axial Length Changes and Myopia in Children and Adolescents
Source: Nutrients. 2026 Jul 16;18(14):2326. doi: 10.3390/nu18142326 (PMC13414676; doi:10.3390/nu18142326)
Supplement: Supplementary file 1 [file nutrients-18-02326-s001.zip › nutrients-4403482-supplementary.pdf]

**Consumption of beverages is associated with refractive changes, axial length changes, and myopia in children and adolescents**

***Keyang Zheng*** [a, 1]***, Dongling Yang*** [a, 1]***, Xiaodong Sun*** [a]***, Shuangxiao Qu*** [a]***, Liting Chu*** [a]***, Shenglei Huang*** [a]***, Yuting Huang*** [a]***, Yanting Yang*** [a]***, Wenjuan Qi*** [a]***, Fengyun Zhang*** [a, 1, *]***, and Chunyan Luo*** [a, 1, *]

[a] Shanghai Municipal Center for Disease Control and Prevention, Division of Child and Adolescent Health, Shanghai 200336, China

[1] These authors contributed equally to this study

[*] Corresponding author

*Email: luochunyan@scdc.sh.cn; zhangfengyun@scdc.sh.cn.*

**Table and Figure of Contents**

**Table S1**. Generalized estimating equations analysis of the frequency of consumption of various beverages and myopia among research participants from 2021 to 2024 (n = 5199) after adjusting for parental myopia.

**Table S2**. Generalized estimating equations analysis of the frequency of consumption of various beverages and spherical equivalent (SE) among research participants from 2021 to 2024 (n = 5199) after adjusting for parental myopia.

**Table S3**. Generalized estimating equations analysis of the frequency of consumption of various beverages and axial length (AL) among research participants from 2021 to 2024 (n = 4751) after adjusting for parental myopia.

**Table S4**. Generalized estimating equations analysis of the frequency of consumption of various beverages and myopia among research participants from 2021 to 2024 (n = 5199) after adjusting for eye-use habits.

**Table S5**. Generalized estimating equations analysis of the frequency of consumption of various beverages and spherical equivalent (SE) among research participants from 2021 to 2024 (n = 5199) after adjusting for eye-use habits.

**Table S6**. Generalized estimating equations analysis of the frequency of consumption of various beverages and axial length (AL) among research participants from 2021 to 2024 (n = 4751) after adjusting for eye-use habits.

**Figure S1**. Flowchart for the inclusion of students from Shanghai 16 districts in this study.

**Figure S2.** Directed acyclic graph for the association between sugar-sweetened beverages and visual impairment in children.

**Table S1.** Generalized estimating equations analysis of the frequency of consumption of various beverages and myopia among research participants from 2021 to 2024 (n = 5199) after adjusting for parental myopia.

| Variable | Group | Left eye | | Right eye | | Overall | |
|---|---|---|---|---|---|---|---|
| | | OR (95%CI) | *p* | OR (95%CI) | *p* | OR (95%CI) | *p* |
| 100% fruit and vegetable juice (times/week) | Never | Ref | - | Ref | - | Ref | - |
| | ≤1 | 0.779 (0.711, 0.852) | <0.001 | 0.745 (0.680, 0.815) | <0.001 | 0.751 (0.687, 0.821) | <0.001 |
| | ＞1 to ≤3 | 0.776 (0.706, 0.854) | <0.001 | 0.738 (0.671, 0.813) | <0.001 | 0.750 (0.682, 0.825) | <0.001 |
| | ＞3 | 0.869 (0.774, 0.976) | 0.017 | 0.836 (0.746, 0.938) | 0.002 | 0.825 (0.736, 0.925) | 0.001 |
| Non-100% fruit and vegetable juice (times/week) | Never | Ref | - | Ref | - | Ref | - |
| | ≤1 | 0.808 (0.736, 0.889) | <0.001 | 0.760 (0.692, 0.836) | <0.001 | 0.757 (0.690, 0.831) | <0.001 |
| | ＞1 to ≤3 | 0.867 (0.771, 0.975) | 0.017 | 0.856 (0.761, 0.962) | 0.009 | 0.881 (0.783, 0.990) | 0.033 |
| | ＞3 | 1.040 (0.886, 1.221) | 0.631 | 1.068 (0.910, 1.254) | 0.421 | 1.074 (0.914, 1.261) | 0.387 |
| Carbonated beverages | Never | Ref | - | Ref | - | Ref | - |

| | | | | | | | |
|---|---|---|---|---|---|---|---|
| (times/week) | | | | | | | |
| | ≤1 | 1.042 (0.952, 1.142) | 0.370 | 1.075 (0.982, 1.176) | 0.118 | 1.076 (0.983, 1.177) | 0.111 |
| | ＞1 to ≤3 | 1.104 (0.972, 1.254) | 0.129 | 1.127 (0.992, 1.280) | 0.066 | 1.128 (0.991, 1.283) | 0.068 |
| | ＞3 | 1.273 (1.053, 1.538) | 0.013 | 1.377 (1.141, 1.663) | 0.001 | 1.418 (1.173, 1.713) | <0.001 |
| Milk tea beverages (times/week) | Never | Ref | - | Ref | - | Ref | - |
| | ≤1 | 1.494 (1.370, 1.630) | <0.001 | 1.489 (1.365, 1.623) | <0.001 | 1.514 (1.388, 1.651) | <0.001 |
| | ＞1 to ≤3 | 2.005 (1.716, 2.342) | <0.001 | 2.013 (1.715, 2.362) | <0.001 | 2.038 (1.736, 2.393) | <0.001 |
| | ＞3 | 1.314 (1.061, 1.628) | 0.012 | 1.205 (0.975, 1.489) | 0.084 | 1.266 (1.024, 1.564) | 0.029 |
| Other sugary beverages [a] (times/week) | Never | Ref | - | Ref | - | Ref | - |
| | ≤1 | 0.904 (0.829, 0.985) | 0.022 | 0.944 (0.867, 1.029) | 0.191 | 0.907 (0.832, 0.989) | 0.027 |
| | ＞1 to ≤3 | 0.900 (0.811, 0.999) | 0.048 | 0.908 (0.818, 1.007) | 0.068 | 0.877 (0.790, 0.974) | 0.014 |
| | ＞3 | 0.989 (0.862, 1.135) | 0.874 | 0.959 (0.836, 1.100) | 0.551 | 0.960 (0.837, 1.101) | 0.562 |
| Non-sugar-sweetened beverages [b] (times/week) | Never | Ref | - | Ref | - | Ref | - |

| | | | | | | | |
|---|---|---|---|---|---|---|---|
| | ≤1 | 1.000 (0.895, 1.116) | 0.998 | 0.997 (0.894, 1.111) | 0.951 | 1.001 (0.898, 1.116) | 0.987 |
| | ＞1 to ≤3 | 1.217 (0.999, 1.484) | 0.052 | 1.285 (1.048, 1.576) | 0.016 | 1.318 (1.078, 1.612) | 0.007 |
| | ＞3 | 1.076 (0.835, 1.387) | 0.572 | 1.158 (0.899, 1.492) | 0.257 | 1.119 (0.868, 1.441) | 0.386 |
| Paternal myopia | No | Ref | - | Ref | - | Ref | - |
| | Yes | 1.352 (1.230, 1.487) | <0.001 | 1.333 (1.213, 1.464) | <0.001 | 1.322 (1.205, 1.451) | <0.001 |
| Maternal myopia | No | Ref | - | Ref | - | Ref | - |
| | Yes | 1.135 (1.032, 1.249) | 0.009 | 1.187 (1.080, 1.305) | <0.001 | 1.135 (1.034, 1.246) | 0.008 |

Note: OR, odds ratio; CI, confidence interval; Ref, reference. The data show the consumption of various beverages within the month prior to the survey date. The model adjusted for children's age, gender, ethnicity, body mass index (BMI), parents' education level, household economic status, participation in extracurricular sports courses, daily outdoor activity duration on school days, outdoor activity duration on weekends, urban-rural type, and parental myopia.

[a]: Including tea-flavored beverages, dairy-containing or lactic acid bacteria beverages, plant protein and grain beverages, coffee, and functional beverages; [b]: Drinks labeled as sugar-free but with added sweeteners.

**Table S2.** Generalized estimating equations analysis of the frequency of consumption of various beverages and spherical equivalent (SE) among research participants from 2021 to 2024 (n = 5199) after adjusting for parental myopia.

| Variable | Group | Left eye | | Right eye | |
|---|---|---|---|---|---|
| | | β (95%CI) | *p* | β (95%CI) | *p* |
| 100% fruit and vegetable juice (times/week) | Never | Ref | - | Ref | - |
| | ≤1 | 0.278 (0.195, 0.361) | <0.001 | 0.296 (0.212, 0.380) | <0.001 |
| | ＞1 to ≤3 | 0.298 (0.209, 0.386) | <0.001 | 0.324 (0.235, 0.413) | <0.001 |
| | ＞3 | 0.227 (0.118, 0.336) | <0.001 | 0.239 (0.130, 0.349) | <0.001 |
| Non-100% fruit and vegetable juice (times/week) | Never | Ref | - | Ref | - |
| | ≤1 | 0.221 (0.137, 0.304) | <0.001 | 0.252 (0.166, 0.338) | <0.001 |
| | ＞1 to ≤3 | 0.162 (0.054, 0.269) | 0.003 | 0.207 (0.098, 0.315) | <0.001 |
| | ＞3 | -0.029 (-0.182, 0.123) | 0.706 | -0.033 (-0.185, 0.120) | 0.675 |
| Carbonated beverages (times/week) | Never | Ref | - | Ref | - |
| | ≤1 | -0.006 (-0.095, 0.082) | 0.887 | -0.020 (-0.109, 0.069) | 0.660 |

| | | | | | |
|---|---|---|---|---|---|
| | ＞1 to ≤3 | -0.072 (-0.202, 0.058) | 0.280 | -0.109 (-0.241, 0.023) | 0.107 |
| | ＞3 | -0.260 (-0.453, -0.067) | 0.008 | -0.220 (-0.414, -0.026) | 0.026 |
| Milk tea beverages (times/week) | Never | Ref | - | Ref | - |
| | ≤1 | -0.409 (-0.496, -0.321) | <0.001 | -0.464 (-0.551, -0.377) | <0.001 |
| | ＞1 to ≤3 | -0.705 (-0.868, -0.541) | <0.001 | -0.731 (-0.902, -0.561) | <0.001 |
| | ＞3 | -0.254 (-0.475, -0.033) | 0.024 | -0.268 (-0.495, -0.041) | 0.020 |
| Other sugary beverages [a] (times/week) | Never | Ref | - | Ref | - |
| | ≤1 | 0.092 (0.010, 0.174) | 0.028 | 0.124 (0.041, 0.208) | 0.004 |
| | ＞1 to ≤3 | 0.079 (-0.020, 0.178) | 0.119 | 0.123 (0.023, 0.223) | 0.016 |
| | ＞3 | -0.049 (-0.186, 0.088) | 0.483 | -0.033 (-0.171, 0.106) | 0.644 |
| Non-sugar-sweetened beverages [b] (times/week) | Never | Ref | - | Ref | - |
| | ≤1 | -0.013 (-0.118, 0.092) | 0.811 | -0.012 (-0.118, 0.094) | 0.823 |
| | ＞1 to ≤3 | -0.202 (-0.390, -0.014) | 0.035 | -0.192 (-0.378, -0.005) | 0.044 |
| | ＞3 | -0.164 (-0.431, 0.103) | 0.229 | -0.218 (-0.490, 0.054) | 0.116 |
| Paternal myopia | No | Ref | - | Ref | - |
| | Yes | -0.340 (-0.442, -0.239) | <0.001 | -0.329 (-0.431, -0.227) | <0.001 |

| | | | | | |
|---|---|---|---|---|---|
| Maternal myopia | No | Ref | - | Ref | - |
| | Yes | -0.153 (-0.254, -0.051) | 0.003 | -0.142 (-0.245, -0.040) | 0.006 |

Note: β, regression coefficient; CI, confidence interval; Ref, reference. The data show the consumption of various beverages within the month prior to the survey date. The model adjusted for children's age, gender, ethnicity, body mass index (BMI), parents' education level, household economic status, participation in extracurricular sports courses, daily outdoor activity duration on school days, outdoor activity duration on weekends, urban-rural type, and parental myopia.

[a]: Including tea-flavored beverages, dairy-containing or lactic acid bacteria beverages, plant protein and grain beverages, coffee, and functional beverages; [b]: Drinks labeled as sugar-free but with added sweeteners.

**Table S3.** Generalized estimating equations analysis of the frequency of consumption of various beverages and axial length (AL) among research participants from 2021 to 2024 (n = 4751) after adjusting for parental myopia.

| Variable | Group | Left eye | | Right eye | |
|---|---|---|---|---|---|
| | | β (95%CI) | *p* | β (95%CI) | *p* |
| 100% fruit and vegetable juice (times/week) | Never | Ref | - | Ref | - |
| | ≤1 | -0.180 (-0.246, -0.114) | <0.001 | -0.206 (-0.272, -0.140) | <0.001 |
| | ＞1 to ≤3 | -0.191 (-0.260, -0.122) | <0.001 | -0.202 (-0.272, -0.133) | <0.001 |
| | ＞3 | -0.097 (-0.181, -0.012) | 0.025 | -0.105 (-0.189, -0.022) | 0.014 |
| Non-100% fruit and vegetable juice (times/week) | Never | Ref | - | Ref | - |
| | ≤1 | -0.186 (-0.253, -0.119) | <0.001 | -0.193 (-0.261, -0.126) | <0.001 |
| | ＞1 to ≤3 | -0.126 (-0.210, -0.043) | 0.003 | -0.117 (-0.202, -0.032) | 0.007 |
| | ＞3 | -0.063 (-0.179, 0.053) | 0.286 | -0.063 (-0.177, 0.052) | 0.283 |
| Carbonated beverages (times/week) | Never | Ref | - | Ref | - |
| | ≤1 | 0.053 (-0.014, 0.119) | 0.120 | 0.045 (-0.022, 0.111) | 0.189 |

| | | | | | |
|---|---|---|---|---|---|
| | ＞1 to ≤3 | 0.228 (0.132, 0.324) | <0.001 | 0.237 (0.138, 0.337) | <0.001 |
| | ＞3 | 0.391 (0.260, 0.523) | <0.001 | 0.384 (0.254, 0.514) | <0.001 |
| Milk tea beverages (times/week) | Never | Ref | - | Ref | - |
| | ≤1 | 0.160 (0.096, 0.224) | <0.001 | 0.174 (0.110, 0.238) | <0.001 |
| | ＞1 to ≤3 | 0.319 (0.203, 0.435) | <0.001 | 0.311 (0.191, 0.431) | <0.001 |
| | ＞3 | -0.024 (-0.197, 0.148) | 0.781 | -0.039 (-0.211, 0.133) | 0.654 |
| Other sugary beverages [a] (times/week) | Never | Ref | - | Ref | - |
| | ≤1 | -0.039 (-0.101, 0.023) | 0.220 | -0.049 (-0.112, 0.014) | 0.126 |
| | ＞1 to ≤3 | 0.005 (-0.074, 0.084) | 0.899 | 0.004 (-0.075, 0.084) | 0.915 |
| | ＞3 | 0.052 (-0.047, 0.151) | 0.305 | 0.024 (-0.075, 0.123) | 0.634 |
| Non-sugar-sweetened beverages [b] (times/week) | Never | Ref | - | Ref | - |
| | ≤1 | 0.054 (-0.026, 0.133) | 0.187 | 0.063 (-0.016, 0.141) | 0.119 |
| | ＞1 to ≤3 | 0.122 (-0.018, 0.262) | 0.088 | 0.140 (-0.003, 0.283) | 0.055 |
| | ＞3 | 0.156 (-0.036, 0.348) | 0.111 | 0.182 (-0.010, 0.375) | 0.063 |
| Paternal myopia | No | Ref | - | Ref | - |
| | Yes | 0.101 (0.030, 0.172) | 0.006 | 0.099 (0.027, 0.170) | 0.007 |

| | | | | | |
|---|---|---|---|---|---|
| Maternal myopia | No | Ref | - | Ref | - |
| | Yes | 0.018 (-0.053, 0.089) | 0.623 | 0.002 (-0.070, 0.073) | 0.966 |

Note: β, regression coefficient; CI, confidence interval; Ref, reference. The data show the consumption of various beverages within the month prior to the survey date. The model adjusted for children's age, gender, ethnicity, body mass index (BMI), parents' education level, household economic status, participation in extracurricular sports courses, daily outdoor activity duration on school days, outdoor activity duration on weekends, urban-rural type, and parental myopia .

[a]: Including tea-flavored beverages, dairy-containing or lactic acid bacteria beverages, plant protein and grain beverages, coffee, and functional beverages; [b]: Drinks labeled as sugar-free but with added sweeteners.

**Table S4.** Generalized estimating equations analysis of the frequency of consumption of various beverages and myopia among research participants from 2021 to 2024 (n = 5199) after adjusting for child eye-use habits.

| Variable | Group | Left eye | | Right eye | | Overall | |
|---|---|---|---|---|---|---|---|
| | | OR (95%CI) | *p* | OR (95%CI) | *p* | OR (95%CI) | *p* |
| 100% fruit and vegetable juice (times/week) | Never | Ref | - | Ref | - | Ref | - |
| | ≤1 | 0.821 (0.749, 0.899) | <0.001 | 0.788 (0.719, 0.863) | <0.001 | 0.793 (0.724, 0.869) | <0.001 |
| | ＞1 to ≤3 | 0.826 (0.751, 0.910) | <0.001 | 0.788 (0.715, 0.868) | <0.001 | 0.800 (0.727, 0.880) | <0.001 |
| | ＞3 | 0.884 (0.787, 0.993) | 0.037 | 0.850 (0.757, 0.954) | 0.006 | 0.839 (0.748, 0.941) | 0.003 |
| Non-100% fruit and vegetable juice (times/week) | Never | Ref | - | Ref | - | Ref | - |
| | ≤1 | 0.819 (0.745, 0.901) | <0.001 | 0.769 (0.699, 0.845) | <0.001 | 0.766 (0.697, 0.841) | <0.001 |
| | ＞1 to ≤3 | 0.855 (0.759, 0.962) | 0.010 | 0.843 (0.749, 0.949) | 0.005 | 0.868 (0.771, 0.978) | 0.020 |
| | ＞3 | 0.992 (0.843, 1.168) | 0.925 | 1.015 (0.861, 1.196) | 0.863 | 1.023 (0.867, 1.207) | 0.786 |
| Carbonated beverages | Never | Ref | - | Ref | - | Ref | - |

| | | | | | | | |
|---|---|---|---|---|---|---|---|
| (times/week) | | | | | | | |
| | ≤1 | 1.047 (0.955, 1.148) | 0.325 | 1.081 (0.987, 1.183) | 0.093 | 1.081 (0.987, 1.183) | 0.094 |
| | ＞1 to ≤3 | 1.064 (0.936, 1.209) | 0.344 | 1.087 (0.956, 1.235) | 0.203 | 1.086 (0.954, 1.236) | 0.213 |
| | ＞3 | 1.174 (0.967, 1.425) | 0.105 | 1.270 (1.048, 1.539) | 0.015 | 1.312 (1.081, 1.592) | 0.006 |
| Milk tea beverages (times/week) | Never | Ref | - | Ref | - | Ref | - |
| | ≤1 | 1.417 (1.297, 1.547) | <0.001 | 1.407 (1.289, 1.535) | <0.001 | 1.432 (1.312, 1.564) | <0.001 |
| | ＞1 to ≤3 | 1.810 (1.548, 2.117) | <0.001 | 1.808 (1.539, 2.124) | <0.001 | 1.832 (1.558, 2.153) | <0.001 |
| | ＞3 | 1.255 (1.012, 1.558) | 0.039 | 1.149 (0.928, 1.422) | 0.203 | 1.213 (0.980, 1.501) | 0.076 |
| Other sugary beverages [a] (times/week) | Never | Ref | - | Ref | - | Ref | - |
| | ≤1 | 0.912 (0.836, 0.995) | 0.039 | 0.956 (0.876, 1.042) | 0.303 | 0.916 (0.839, 0.999) | 0.047 |
| | ＞1 to ≤3 | 0.904 (0.814, 1.003) | 0.058 | 0.912 (0.822, 1.013) | 0.085 | 0.879 (0.791, 0.977) | 0.016 |
| | ＞3 | 1.005 (0.874, 1.156) | 0.940 | 0.979 (0.851, 1.125) | 0.761 | 0.978 (0.851, 1.125) | 0.755 |
| Non-sugar-sweetened beverages [b] (times/week) | Never | Ref | - | Ref | - | Ref | - |

| | | | | | | | |
|---|---|---|---|---|---|---|---|
| | ≤1 | 0.991 (0.887, 1.108) | 0.878 | 0.987 (0.885, 1.101) | 0.814 | 0.993 (0.890, 1.108) | 0.901 |
| | ＞1 to ≤3 | 1.222 (1.001, 1.493) | 0.049 | 1.291 (1.052, 1.585) | 0.015 | 1.325 (1.083, 1.622) | 0.006 |
| | ＞3 | 1.092 (0.846, 1.410) | 0.498 | 1.178 (0.913, 1.520) | 0.208 | 1.135 (0.880, 1.464) | 0.328 |
| Daily reading time (hour/day) | ≤1 | Ref | - | Ref | - | Ref | - |
| | ＞1 | 1.413 (1.276, 1.566) | <0.001 | 1.445 (1.305, 1.600) | <0.001 | 1.471 (1.331, 1.626) | <0.001 |
| Daily screen time (hour/day) | ≤1 | Ref | - | Ref | - | Ref | - |
| | ＞1 | 1.124 (1.016, 1.243) | 0.024 | 1.098 (0.994, 1.214) | 0.066 | 1.111 (1.006, 1.227) | 0.038 |

Note: OR, odds ratio; CI, confidence interval; Ref, reference. The data show the consumption of various beverages within the month prior to the survey date. The model adjusted for children's age, gender, ethnicity, body mass index (BMI), parents' education level, household economic status, participation in extracurricular sports courses, daily outdoor activity duration on school days, outdoor activity duration on weekends, urban-rural type, and child eye-use habits.

[a]: Including tea-flavored beverages, dairy-containing or lactic acid bacteria beverages, plant protein and grain beverages, coffee, and functional beverages; [b]: Drinks labeled as sugar-free but with added sweeteners.

**Table S5.** Generalized estimating equations analysis of the frequency of consumption of various beverages and spherical equivalent (SE) among research participants from 2021 to 2024 (n = 5199) after adjusting for child eye-use habits.

| Variable | Group | Left eye | | Right eye | |
|---|---|---|---|---|---|
| | | β (95%CI) | *p* | β (95%CI) | *p* |
| 100% fruit and vegetable juice (times/week) | Never | Ref | - | Ref | - |
| | ≤1 | 0.218 (0.135, 0.301) | <0.001 | 0.231 (0.148, 0.314) | <0.001 |
| | ＞1 to ≤3 | 0.228 (0.140, 0.315) | <0.001 | 0.249 (0.161, 0.338) | <0.001 |
| | ＞3 | 0.208 (0.100, 0.315) | <0.001 | 0.217 (0.109, 0.325) | <0.001 |
| Non-100% fruit and vegetable juice (times/week) | Never | Ref | - | Ref | - |
| | ≤1 | 0.204 (0.121, 0.287) | <0.001 | 0.234 (0.150, 0.319) | <0.001 |
| | ＞1 to ≤3 | 0.173 (0.066, 0.280) | 0.002 | 0.218 (0.110, 0.325) | <0.001 |
| | ＞3 | 0.021 (-0.130, 0.172) | 0.786 | 0.019 (-0.133, 0.170) | 0.808 |
| Carbonated beverages (times/week) | Never | Ref | - | Ref | - |
| | ≤1 | -0.011 (-0.099, 0.077) | 0.803 | -0.024 (-0.111, 0.064) | 0.598 |

| | | | | | |
|---|---|---|---|---|---|
| | ＞1 to ≤3 | -0.036 (-0.166, 0.094) | 0.588 | -0.070 (-0.201, 0.061) | 0.296 |
| | ＞3 | -0.178 (-0.373, 0.017) | 0.074 | -0.133 (-0.329, 0.062) | 0.182 |
| Milk tea beverages (times/week) | Never | Ref | - | Ref | - |
| | ≤1 | -0.345 (-0.432, -0.257) | <0.001 | -0.395 (-0.481, -0.308) | <0.001 |
| | ＞1 to ≤3 | -0.590 (-0.752, -0.428) | <0.001 | -0.607 (-0.775, -0.439) | <0.001 |
| | ＞3 | -0.199 (-0.421, 0.023) | 0.079 | -0.214 (-0.441, 0.013) | 0.065 |
| Other sugary beverages [a] (times/week) | Never | Ref | - | Ref | - |
| | ≤1 | 0.079 (-0.003, 0.161) | 0.058 | 0.113 (0.030, 0.196) | 0.008 |
| | ＞1 to ≤3 | 0.073 (-0.026, 0.172) | 0.147 | 0.118 (0.020, 0.217) | 0.019 |
| | ＞3 | -0.070 (-0.207, 0.066) | 0.311 | -0.054 (-0.191, 0.084) | 0.443 |
| Non-sugar-sweetened beverages [b] (times/week) | Never | Ref | - | Ref | - |
| | ≤1 | -0.005 (-0.109, 0.099) | 0.926 | -0.004 (-0.109, 0.100) | 0.935 |
| | ＞1 to ≤3 | -0.208 (-0.395, -0.022) | 0.029 | -0.196 (-0.381, -0.011) | 0.038 |
| | ＞3 | -0.176 (-0.444, 0.092) | 0.199 | -0.229 (-0.501, 0.042) | 0.098 |
| Daily reading time (hour/day) | ≤1 | Ref | - | Ref | - |
| | ＞1 | -0.277 (-0.383, -0.170) | <0.001 | -0.328 (-0.437, -0.220) | <0.001 |

| | | | | | |
|---|---|---|---|---|---|
| Daily screen time (hour/day) | ≤1 | Ref | - | Ref | - |
| | ＞1 | -0.108 (-0.219, 0.002) | 0.054 | -0.120 (-0.229, -0.010) | 0.032 |

Note: β, regression coefficient; CI, confidence interval; Ref, reference. The data show the consumption of various beverages within the month prior to the survey date. The model adjusted for children's age, gender, ethnicity, body mass index (BMI), parents' education level, household economic status, participation in extracurricular sports courses, daily outdoor activity duration on school days, outdoor activity duration on weekends, urban-rural type and child eye-use habits.

[a]: Including tea-flavored beverages, dairy-containing or lactic acid bacteria beverages, plant protein and grain beverages, coffee, and functional beverages; [b]: Drinks labeled as sugar-free but with added sweeteners.

**Table S6.** Generalized estimating equations analysis of the frequency of consumption of various beverages and axial length (AL) among research participants from 2021 to 2024 (n = 4751) after adjusting for child eye-use habits.

| Variable | Group | Left eye | | Right eye | |
|---|---|---|---|---|---|
| | | β (95%CI) | *p* | β (95%CI) | *p* |
| 100% fruit and vegetable juice (times/week) | Never | Ref | - | Ref | - |
| | ≤1 | -0.143 (-0.208, -0.078) | <0.001 | -0.167 (-0.231, -0.103) | <0.001 |
| | ＞1 to ≤3 | -0.144 (-0.212, -0.075) | <0.001 | -0.152 (-0.220, -0.084) | <0.001 |
| | ＞3 | -0.082 (-0.167, 0.002) | 0.055 | -0.089 (-0.173, -0.006) | 0.036 |
| Non-100% fruit and vegetable juice (times/week) | Never | Ref | - | Ref | - |
| | ≤1 | -0.178 (-0.243, -0.112) | <0.001 | -0.184 (-0.250, -0.118) | <0.001 |
| | ＞1 to ≤3 | -0.128 (-0.211, -0.045) | 0.003 | -0.118 (-0.202, -0.035) | 0.006 |
| | ＞3 | -0.076 (-0.190, 0.039) | 0.195 | -0.075 (-0.189, 0.039) | 0.197 |
| Carbonated beverages (times/week) | Never | Ref | - | Ref | - |
| | ≤1 | 0.051 (-0.015, 0.117) | 0.129 | 0.042 (-0.023, 0.107) | 0.208 |

| | | | | | |
|---|---|---|---|---|---|
| | ＞1 to ≤3 | 0.207 (0.112, 0.302) | <0.001 | 0.214 (0.116, 0.312) | <0.001 |
| | ＞3 | 0.353 (0.223, 0.483) | <0.001 | 0.345 (0.215, 0.474) | <0.001 |
| Milk tea beverages (times/week) | Never | Ref | - | Ref | - |
| | ≤1 | 0.127 (0.064, 0.190) | <0.001 | 0.139 (0.076, 0.202) | <0.001 |
| | ＞1 to ≤3 | 0.257 (0.142, 0.372) | <0.001 | 0.244 (0.126, 0.362) | <0.001 |
| | ＞3 | -0.042 (-0.211, 0.127) | 0.627 | -0.058 (-0.227, 0.111) | 0.503 |
| Other sugary beverages [a] (times/week) | Never | Ref | - | Ref | - |
| | ≤1 | -0.042 (-0.103, 0.019) | 0.176 | -0.054 (-0.115, 0.008) | 0.086 |
| | ＞1 to ≤3 | 0.003 (-0.075, 0.082) | 0.935 | 0.001 (-0.077, 0.080) | 0.974 |
| | ＞3 | 0.069 (-0.029, 0.167) | 0.168 | 0.041 (-0.056, 0.139) | 0.407 |
| Non-sugar-sweetened beverages [b] (times/week) | Never | Ref | - | Ref | - |
| | ≤1 | 0.050 (-0.028, 0.128) | 0.210 | 0.059 (-0.018, 0.137) | 0.132 |
| | ＞1 to ≤3 | 0.131 (-0.009, 0.271) | 0.066 | 0.150 (0.007, 0.292) | 0.039 |
| | ＞3 | 0.148 (-0.041, 0.338) | 0.125 | 0.172 (-0.017, 0.362) | 0.074 |
| Daily reading time (hour/day) | ≤1 | Ref | - | Ref | - |
| | ＞1 | 0.172 (0.097, 0.247) | <0.001 | 0.180 (0.104, 0.255) | <0.001 |

| | | | | | |
|---|---|---|---|---|---|
| Daily screen time (hour/day) | ≤1 | Ref | - | Ref | - |
| | ＞1 | 0.087 (0.012, 0.162) | 0.023 | 0.097 (0.021, 0.172) | 0.012 |

Note: β, regression coefficient; CI, confidence interval; Ref, reference. The data show the consumption of various beverages within the month prior to the survey date. The model adjusted for children's age, gender, ethnicity, body mass index (BMI), parents' education level, household economic status, participation in extracurricular sports courses, daily outdoor activity duration on school days, outdoor activity duration on weekends, urban-rural type and child eye-use habits.

[a]: Including tea-flavored beverages, dairy-containing or lactic acid bacteria beverages, plant protein and grain beverages, coffee, and functional beverages; [b]: Drinks labeled as sugar-free but with added sweeteners.

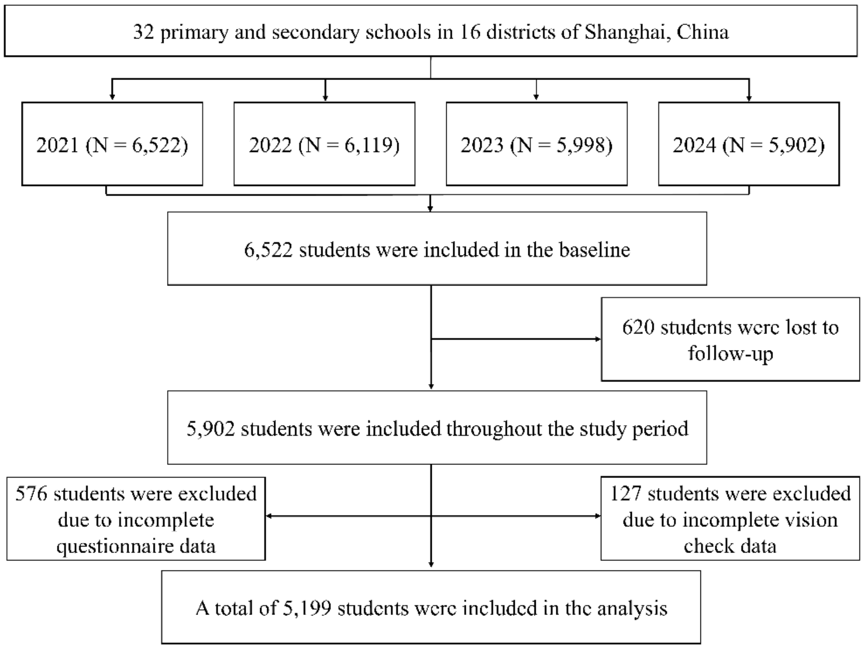


**Figure S1**. Flowchart for the inclusion of students from Shanghai 16 districts in this study.

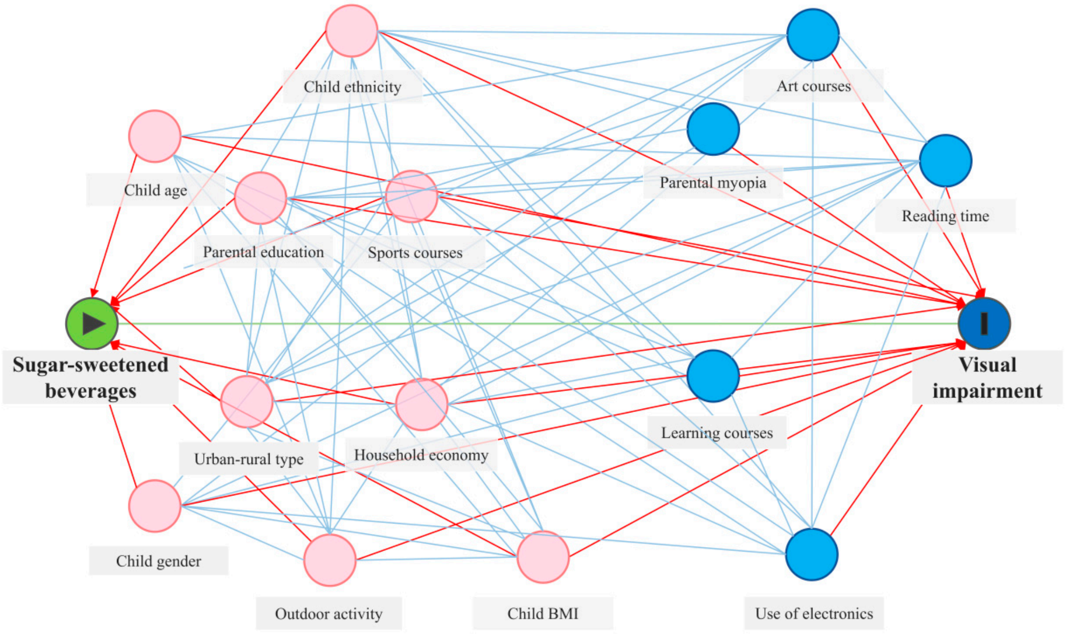


**Figure S2.** Directed acyclic graph for the association between sugar-sweetened beverages and visual impairment in children.
